# Supplementary material for: Translating episodic future thinking manipulations for clinical use: Development of a clinical control
Source: PLoS One. 2020 Aug 18;15(8):e0237435. doi: 10.1371/journal.pone.0237435 (PMC7433876; doi:10.1371/journal.pone.0237435)
Supplement: S1 File — (DOCX) [file pone.0237435.s001.docx]

Please read and consider the following information about **alcohol and sleep**:

Many people swear by the “nightcap” to help them doze off at night. The problem with having a drink before bedtime is, when your body finishes metabolizing the alcohol during the night, you’re likely to wake up (this is sometimes called “rebound alertness”). Drinking alcohol before bed can increase snoring and worsen acid reflux—both of which can impact your quality of sleep. Alcohol also prevents you from slipping into the deeper, more restful stages of sleep. Instead of having alcohol close to bedtime to help make you sleepy, some research suggests tart cherry juice may help induce sleep. Setting a consistent bedtime routine (including putting on a certain type of sleepwear!) helps signal your body that it’s time to rest. But, if you do choose to have an alcoholic drink and still want to get a good night's rest, do so at least three hours before bedtime.

You will be able to progress to the next page momentarily.

Information from “Your Top Sleep Questions Answered” published by Johns Hopkins School of Medicine. Modified for presentation purposes; use for informational purposes only. Retrieved from: <https://www.hopkinsmedicine.org/health/healthy-sleep/download-sleep-guide.html>

Please read and consider the following information about **major depression**:

Major depression is a mood disorder that affects a person’s ability to feel, think, and handle daily activities. While the word “depression” refers to mood, major depression has physical symptoms in addition to the commonly thought of mood-related ones (e.g., persistent sad or empty mood, feelings of worthlessness and pessimism). For instance, feelings of decreased energy, difficulty concentrating, changes in appetite, and digestive problems are also common symptoms of depression. However, just like with other health conditions (e.g., heart attacks), symptoms of depression can vary across men and women. Men with depression are more likely to hide their feelings and may seem to be angry, irritable, or aggressive; whereas many women with depression seem sad or express sadness. Symptoms of depression also vary across the lifespan: children may cling to a parent, pretend to be sick, or worry that a parent may die; and older adults can have less obvious symptoms such as tiredness and attention problems.  

You will be able to progress to the next page momentarily.

Information from brochures “Depression: What You Need To Know” and “Men and Depression” and the corresponding webpages, published by the National Institute of Mental Health. Modified for presentation purposes; use for informational purposes only. Retrieved from https://www.nimh.nih.gov/health/publications/depression-listing.shtml

Please read and consider the following information about **prediabetes**:

Most of the food you eat is broken down into sugar (also called glucose) and released into your bloodstream. Your pancreas makes a hormone called insulin, which acts like a key to let blood sugar into your body’s cells for use as energy. When a person’s body doesn’t respond well to the signal from insulin to let the sugar into cells, sugar can build up in the blood stream and damage the body; this is what happens in Type 2 diabetes. Prediabetes is a similar condition, where the body doesn’t respond to insulin appropriately, and blood sugar levels are higher than normal—although they are not high enough to be diagnosed as type 2 diabetes. But don’t let the “pre” fool you— prediabetes is a serious health condition that can lead to Type 2 diabetes, and it puts you at increased risk of heart disease and stroke. Most who have prediabetes (about 90%!) don’t know they have it. The good news is that if you have prediabetes, you can make lifestyle changes to prevent or delay type 2 diabetes and other serious health problems.


You will be able to progress to the next page momentarily.

Information from “The Surprising Truth About Prediabetes” and “Prediabetes: Your Chance to Prevent Type 2 Diabetes” webpages, published by the Centers for Disease Control and Prevention. Modified for presentation purposes; use for informational purposes only. Retrieved from https://www.cdc.gov/diabetes/basics/prediabetes.html and <https://www.cdc.gov/features/diabetesprevention/index.html>

Please read and consider the following information about **electronic cigarettes**:

Although smoking cigarettes has slowly begun to decline, the introduction of e-cigarettes has produced an increase in the level of nicotine addiction within the population. E-Cigarettes produce an aerosol, instead of smoke, by heating a liquid that usually contains nicotine, flavorings, and other chemicals. E-cigarettes are almost certainly less lethal than conventional cigarettes, but they may still have negative health effects. For instance, chronic exposure to nicotine may lead to insulin resistance and type 2 diabetes, and some liquids used in e-cigarettes contain chemicals linked to the development of rare lung diseases. There is also cause for concern for children and teens using e-cigarettes: nicotine exposure in adolescence can impair prefrontal brain development, which is linked to attention deficit disorder and poor impulse control. These findings have led to the idea that e-cigarettes may be viewed as a means of harm reduction for tobacco cigarette smokers, but that does not mean their use in general is safe.

You will be able to progress to the next page momentarily.

Information from the Harvard Health Blog and the National Institutes of Drug Abuse (a branch of the United States’ National Institutes of Health). Information modified for presentation purposes; use for informational purposes only. 
Retrieved from: 
https://www.drugabuse.gov/publications/drugfacts/electronic-cigarettes-e-cigarettes
<https://www.health.harvard.edu/blog/electronic-cigarettes-good-news-bad-news-2016072510010>

Please read and consider the following information about **nutrition labeling**:

To help U.S. citizens make more informed food choices that support a healthy diet, the Food and Drug Administration (FDA) decided in 2016 to redesign food Nutrition Facts labels. Perhaps one of the biggest changes to the label are the serving sizes: serving sizes must now be based on amounts of foods and beverages that people are actually eating or drinking, not what they should be eating/drinking. For example, Nutrition Facts labels for 20-oz bottles of soda must list the information for the entire bottle—not for an 8 oz serving of soda—because most people tend to drink the entire bottle in one sitting. Other changes include listing the added sugars (both in grams and percent daily value) in a food or beverage: scientific data shows that it is difficult to meet nutrient needs while staying within calorie limits if you consume more than 10 percent of your total daily calories from added sugar. These changes, in addition to others, will help people more easily evaluate how healthy their food options are.

You will be able to progress to the next page momentarily.

Information from the web page “Changes to the Nutrition Facts Label” published by the United States Food and Drug Administration. Modified for presentation purposes; use for informational purposes only. 
Retrieved from https://www.fda.gov/Food/GuidanceRegulation/GuidanceDocumentsRegulatoryInformation/LabelingNutrition/ucm385663.htm

Please read and consider the following information about **physical activity**:

The U.S. Department of Health and Human Services initially suggested that people need to engage in moderate-to-vigorous physical activity (activity that is similar to, or greater in intensity than a brisk walk) in bouts of 10 minutes in order for the activity to have a positive and noticeable impact on health. However, research now indicates that Americans can benefit from smaller durations of moderate-to-vigorous physical activity throughout the day. For example, a brief episode of physical activity like climbing up a few flights of stairs is beneficial, both for general health and brain health. Some benefits of physical activity on brain health occur immediately after moderate-to-vigorous physical activity. For example, immediately after moderate-to-vigorous activity, people experience reduced feelings of state anxiety (short-term anxiety), improved sleep, and improved aspects of cognitive function (memory, attention, ability to plan, etc.).


You will be able to progress to the next page momentarily.

Information from “Physical Activity Guidelines” (second edition) published by the United States Office of Disease Prevention and Health Promotion, and the corresponding website. Modified for presentation purposes; use for informational purposes only. Retrieved from https://health.gov/paguidelines/second-edition/
