# Supplementary material for: Translating episodic future thinking manipulations for clinical use: Development of a clinical control
Source: PLoS One. 2020 Aug 18;15(8):e0237435. doi: 10.1371/journal.pone.0237435 (PMC7433876; doi:10.1371/journal.pone.0237435)
Supplement: Attachment — Submitted filename: EFT Controls - Response to Reviewers.docx [file pone.0237435.s002.docx]

**Reviewer #1**

Introduction:
-In summarizing control conditions against EFT, it may be worth citing work from Benoit and colleagues who have used an ‘estimate’ control condition against EFT. Citing this work would situate the current manuscript in the appropriate literature context.

*Thank you for this suggestion. A brief discussion of the control procedure used in Benoit and colleague’s work is now incorporated into the introduction on page 3.*

Method:
-While not a flaw of the manuscript, it struck me that the health control condition against EFT involves very different cognitive processes. For example, the health control condition by and large involves reading novel (not necessarily positive) information whereas EFT involves generating positive information. Has any work investigated whether generation of EFT is necessary? Would just reading specific future events from other participants have the same downstream consequences as generating specific future events? This could be cited as an avenue for future research to identify mechanisms of change involved in EFT vs. various controls.

*This is a great question, and we have wondered about this ourselves (whether generation of EFT events is the more necessary portion of the manipulation). We do not know any studies that have directly examined this question. Some research has examined the issue of emotional valence in the context of EFT, and across studies it is unclear if EFT needs to be positive for reductions in discounting (e.g., Lin & Epstein, 2014; cf. Liu, Feng, Chen, & Li. 2013). In attempts to maintain the flow of the discussion while honoring the spirit of this comment, we have taken care to underscore the importance in designing control groups and the implications this can have (pgs. 20-21).*

Results:
-The sample had some caveats. For example, 30% of the sample had done EFT/ERT before, and 50% of the sample had done discounting tasks before. The investigators note that these data points are not flaws, and that previous discounting behavior doesn’t impact current discounting behavior. I thought motivation could be stronger here by citing empirical pieces that support the investigators’ claims. I also think the discussion should note that the sample was not completely naïve to study design or tasks.

*This concern is understandable and we appreciate bringing it to our attention. The percentages for prior experience are for the sample as a whole; when applying the percentages to the group ns, the number of participants represented in these sub-groups for prior EFT/ERT experience becomes fairly small (see following breakdown).*

|  |  | Group |  |
| --- | --- | --- | --- |
|  | EFT | ERT | HIT |
|  | n = 62 | n = 71 | n = 65 |
| Prev DD |  |  |  |
| Yes | 34 (54.8%) | 40 (56.3%) | 40 (61.5%) |
| No | 27 (43.5%) | 30 (42.3%) | 25 (38.5%) |
| N/A | 1 (1.6%) | 1 (1.4%) | 0 (0%) |
|  |  |  |  |
| Prev EFT |  |  |  |
| Yes | 14 (22.6%) | 20 (28.2%) | 21 (32.3%) |
| No | 46 (74.2%) | 51 (71.8%) | 44 (67.7%) |
| N/A | 2 (3.2%) | 0 (0%) | 0 (0%) |

*Given that we are assessing discounting in the context of a manipulation, it would seem most appropriate to assess the relation between experience and discounting on a within-group basis in the event it interacts with the independent variable. We conducted Wilcoxon rank-sum tests to evaluate the effects. The p-values for each of these tests are provided in the following table:*

|  | Prev.DD | Prev.EFT |
| --- | --- | --- |
| EFT | 0.51 | 0.62 |
| ERT | 0.25 | 0.15 |
| HIT | 0.84 | 0.69 |

*The results of these comparisons indicate that prior task and manipulation experience are not associated with degree of discounting; and as such, we chose not to include these experience variables in analyses. We previously opted not to include these tests/their results in the manuscript because we did not wish to draw undue attention to them; while we feel we did our due diligence in assessing whether these could be a problematic influence for our manipulation, we do not feel any firm conclusions can be made about task/manipulation experience directly as we did not also assess other variables that may be of importance to that question (e.g., whether someone completed EFT or ERT, how long ago they did so, etc.).*

*We now include some more detail on these tests in the manuscript (pg. 15), and have made sure the corresponding analysis code is included in the R-markdown file.*

Discussion:
-The abstract cites ‘non-specific’ treatment factors as related to the health control. What are the non-specific factors? I thought this line of thinking could’ve played a more prominent role in the discussion section.

*We have clarified our use of non-specific treatment factors in the abstract and have worked to unpack this term a bit more in the introduction section (in part, also in response to one of Reviewer 2’s points).*

**Reviewer #2:**

I think more background is needed on why the ERT procedure is problematic. For instance, it was not clear to me why it was so burdensome to regenerate events and cues repeatedly. More generally, a clearer description of the EFT and ERT procedures earlier would help better situate the need for a novel control condition, as would a little more information on how the EFT is used as an intervention and its theoretical mechanisms of change.

*After re-reading this section of the manuscript, we agree that more background on the procedures in the introduction is helpful in justifying the need for alternate controls. This background info has been incorporated (pgs. 3-4), in addition to further detail on why cue regeneration with ERT would be burdensome/problematic (pgs. 4-5).*

*As it is conceptualized in the literature, some say that EFT broadens temporal horizon, which is putatively responsible for its discounting-reducing effects (e.g., Snider et al. 2016). Subsequent research has not found support for this mechanism (Rung & Madden, 2019), and there has been little other investigation of its theoretical mechanisms of change in discounting. As for health behaviors, changes in discounting are perceived to be the driving force, yet this has not been empirically investigated to the best of our knowledge. Because of the lack of clear knowledge in this area, we have opted not to focus on it extensively in the paper.*

Analogously, more information was needed about what EFT task/intervention features the HIT controls for more effectively than the ERT controls for because this was not obvious to me.

*The HIT procedure is designed to be structurally similar to EFT cue generation while (a) mitigating issues with attempting to control for temporal proximity, (b) reducing the likelihood that the content will engage the neural mechanisms involved in EFT effects, and (c) increasing the perceived connection between the control content and health behaviors. In these ways we believe HIT to be superior to ERT for a clinical context, but we do not believe it controls for theoretical aspects more effectively than ERT (and it was not designed to do so).*

*Because we assessed the performance of the HIT procedure in the context of discounting, and not a targeted health behavior, we understand and agree that why the HIT procedure is a better candidate for clinical settings is not as transparent. Ultimately, this is a proof of concept study that helps illustrate whether such control procedure content could inadvertently influence discounting, which is one of EFT’s hypothesized mechanisms of health behavior change.*

*We have made clarifications throughout the introduction in light of the above. In combination with edits to the discussion section (see below), we believe the purpose and rationale for the HIT procedure is clearer.*

I appreciated that the authors conducted a power analysis to determine sample size, but was not totally clear what was meant re. the “magnitude of the EFT effect” – is this referring to a group difference effect on some parameters of the EFT or a result when the EFT is used as an intervention…? My assumption is this referred to the difference in AUC on the discounting task when it had been paired with EFT relative to ERT but I was not sure.

*The above assumption is correct, and we have edited the wording in this section in attempts to make it clearer.*

This is minor but it was not until the Results on p. 13 that I knew what the sample size actually was – typically this is reported in the Abstract and in the Participants section.

*The recruited N has been added to the participants section and abstract.*

It is important to discuss the limitations and implications of using an mTurk sample that is not clinical in any way. It seems hard to evaluate the clinical utility of a paradigm -a stated primary aim of the current study- without using an analogue or clinical sample.

More broadly, before claims about the clinical utility of the HIT control are warranted, the paradigm requires replication in another sample and tests of inter-rater and test-retest reliability and sensitivity to change, etc.

Taken together, I appreciate that the authors are directly evaluating different control conditions and think this is a valuable line of work, but I have concerns about what can reasonably be concluded from the current study as a stand-alone study.

*In hindsight, we feel some of our wording was misleading and agree that we cannot speak to clinical utility per se. We have made edits in several places to more appropriately discuss the study as a test of a control procedure that is more feasible in, and better meets the needs of a clinical/applied context. We are also more diligent in ensuring the reader remembers that this is a proof of concept study using a non-clinical sample. Translational research often begins with non-clinical samples before moving to clinical samples for clinical applications of basic principles. These edits appear throughout pages 21 to 23.*
