# Supplementary material for: Translating episodic future thinking manipulations for clinical use: Development of a clinical control
Source: PLoS One. 2020 Aug 18;15(8):e0237435. doi: 10.1371/journal.pone.0237435 (PMC7433876; doi:10.1371/journal.pone.0237435)
Supplement: Attachment — Submitted filename: EFT Controls - R2 - Response to Rev1.docx [file pone.0237435.s003.docx]

**Reviewer #1**

The abbreviation HIT is used for two different things; perhaps just refer to human intelligence tasks throughout without abbreviating (or use a phrase like “AMT task” or similar) to avoid confusion.

I think it should be “bears structural resemblance” (line 424)

*The above are both excellent catches and we greatly appreciate the keen eye. We have changed the AMT task-related HIT references to just refer to “AMT tasks” or “tasks” where relevant; and the typo has been fixed.*

*While making the above edits, we also realized that the criteria for providing the bonus payment to workers was not described. This is now included after the criteria for awarding base compensation (task approval).*
